# MET Expression in Upper Gastrointestinal Adenocarcinoma: Prevalence, Prognostic Impact, and Implications for Anti‐MET Antibody‐Drug Conjugate Therapy

**DOI:** 10.1002/ijc.70634

**Published:** 2026-07-02

**Authors:** Tillmann Bedau, Thomas Zander, Hans Anton Schlößer, Hakan Alakus, Onur Mustafov, Reinhard Büttner, Christiane Bruns, Alexander Quaas

**Affiliations:** ^1^ Institute of Pathology, Faculty of Medicine and University Hospital Cologne Cologne Germany; ^2^ Department of Internal Medicine Faculty of Medicine and University Hospital Cologne Cologne Germany; ^3^ Department of General, Visceral and Cancer Surgery Faculty of Medicine and University Hospital of Cologne Cologne Germany

**Keywords:** antibody‐drug‐conjugates (ADC), esophageal cancer, gastric cancer, MET‐expression, telisotuzumab vedotin

## Abstract

MET is an actionable receptor tyrosine kinase, and MET‐directed antibody–drug conjugates (ADCs) have recently entered clinical practice with FDA approval in non‐small cell lung cancer and are now being evaluated across gastrointestinal malignancies, with patient selection based on MET immunohistochemistry (IHC) 3+ membranous staining at varying percentage thresholds. However, robust real‐world data on MET protein expression and clinically relevant ADC‐aligned thresholds in upper GI adenocarcinomas remain limited. We profiled 1532 upper GI adenocarcinomas using the clinically validated VENTANA MET (SP44) IHC assay. MET was stratified by (i) 3+ membranous staining fractions (any, ≥ 10%, ≥ 50%) reflecting contemporary ADC eligibility concepts and (ii) *H*‐score (0–300; high ≥ 150). Overall survival analyses and multivariable Cox regression analyses were performed. High MET expression was uncommon (*H*‐score ≥ 150: 2.3%; any 3+: 1.6%; ≥ 10% 3+: 1.3%; ≥ 50% 3+: 1.0%) and enriched in advanced T stage. MET IHC strongly predicted MET amplification (*H*‐score ≥ 150: 63.6% amplified vs. 2.4% in negatives; any 3+: 75.0% vs. 2.6%; all *p* < 0.001). Any MET 3+ staining was independently associated with inferior overall survival (adjusted HR 2.22, 95% CI 1.29–3.83). Most MET 3+ tumors lacked concurrent HER2 positivity, Claudin‐18.2 expression, or dMMR/MSI. In the largest real‐world cohort to date, MET overexpression identifies a rare, biologically aggressive subset with poor outcomes. By applying the latest MET‐ADC–relevant IHC criteria (including the ≥ 10% 3+ threshold), this study provides clinically translatable prevalence estimates and supports standardized MET testing to inform prospective MET‐directed ADC trials in upper GI adenocarcinoma.

## What's New?

MET receptor tyrosine kinase is a driver of tumor progression and metastasis in upper gastrointestinal adenocarcinoma. The prevalence of MET overexpression in gastrointestinal adenocarcinomas, however, remains undefined. In this study, MET protein expression was analyzed in esophageal, gastroesophageal junction, and gastric adenocarcinomas using a validated immunohistochemical assay and relevant MET‐targeted therapy eligibility thresholds. MET overexpression was rare but strongly associated with MET gene amplification, advanced disease, and significantly worse overall survival. Most MET‐positive tumors lacked other actionable biomarkers. The findings identify MET overexpression as a marker of aggressive tumor subtype and support standardized MET testing to guide targeted treatment strategies.

## Graphical abstract

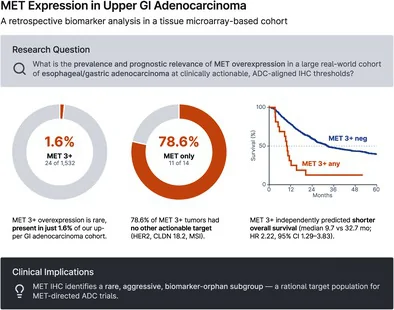

**Abbreviations**
ADC: antibody–drug conjugate
CI: confidence interval
CRC: colorectal cancer
CROSS: chemo‐radiotherapy for oesophageal cancer followed by surgery study
dMMR: mismatch repair‐deficient
EAC: esophageal adenocarcinoma
EGJ: esophagogastric junction
FDA: Food and Drug Administration
FISH: fluorescence in situ hybridization
FLOT: Fluorouracil, Leucovorin, Oxaliplatin and Docetaxel
GAC: gastric adenocarcinoma
GEA: gastric/gastroesophageal junction adenocarcinoma
GEJ: gastroesophageal junction
GI: gastrointestinal
HER2: human epidermal growth factor receptor 2
HGF: hepatocyte growth factor
HR: hazard ratio
IHC: immunohistochemistry
IQR: interquartile range
MAGIC: Medical Research Council Adjuvant Gastric Infusional Chemotherapy
MMR: mismatch repair
MSI: microsatellite instability
MSS: microsatellite stable
NSCLC: non‐small cell lung cancer
OR: odds ratio
pMMR: mismatch repair‐proficient
TMA: tissue microarray

## 1 Introduction

Adenocarcinomas of the upper gastrointestinal (GI) tract, including esophageal adenocarcinoma, adenocarcinoma of the esophagogastric junction (EGJ), and gastric cancer, constitute a significant global health burden due to their high incidence and poor survival outcomes. These tumors collectively account for more than 1 million new cancer diagnoses and deaths worldwide annually, with 5‐year overall survival rates in advanced stages remaining suboptimal despite improvements in multimodal therapy [1, 2, 3]. While gastric cancer incidence has declined in many high‐income regions, esophageal adenocarcinoma and EGJ adenocarcinoma have risen sharply, likely driven by obesity, gastroesophageal reflux disease, and Barrett's esophagus prevalence [2, 4, 5, 6]. The epidemiological shift underscores the growing clinical importance of these tumor subtypes and the need for effective therapeutic strategies. Despite progress in targeted therapies for various malignancies, personalized therapeutic options for upper GI adenocarcinomas remain limited. Currently approved biomarker‐driven treatments—such as HER2‐targeted agents for HER2‐positive gastric cancer, immune checkpoint inhibitors especially effective for microsatellite instability‐high tumors, and therapies targeting Claudin‐18.2—cater to restricted subgroups and often yield modest objective response rates, highlighting an unmet need for broader, biomarker‐guided treatment options [7, 8, 9].

MET, a receptor tyrosine kinase activated by hepatocyte growth factor (HGF), also known as the HGF‐receptor, regulates cell proliferation, survival, and motility in normal tissue homeostasis and cancer progression [10, 11]. Dysregulated MET activity—via overexpression or gene amplification—is implicated in tumorigenesis, invasion, and therapeutic resistance, making it an attractive therapeutic target [12, 13]. Recent advances in antibody–drug conjugates (ADCs) have enabled targeted delivery of cytotoxic payloads to tumors with specific protein expression profiles, offering a promising avenue for precision oncology [14]. In May 2025, the U.S. Food and Drug Administration (FDA) granted accelerated approval to telisotuzumab vedotin (Emrelis), a MET‐directed ADC developed by AbbVie, for adults with locally advanced or metastatic non‐squamous non–small cell lung cancer (NSCLC) exhibiting high MET protein overexpression, defined as immunohistochemical (IHC) 3+ staining in ≥ 50% of tumor cells, as determined by an FDA‐approved diagnostic assay (VENTANA MET [SP44] RxDx) [15]. This approval represents the first ADC targeting MET protein overexpression and was supported by Phase 2 data demonstrating clinically meaningful response rates in heavily pretreated NSCLC patients [16]. Although this indication applies to NSCLC, MET‐targeted ADCs have shown antitumor activity in other solid tumor types. A related agent, telisotuzumab adizutecan (ABBV‐400), which couples the same MET‐targeting antibody to a topoisomerase 1 inhibitor payload, demonstrated promising response rates in patients with metastatic colorectal cancer (CRC) in a phase 1 study (NCT05029882) [17]. This agent is now being evaluated in the randomized phase 3 AndroMETa‐CRC trial (NCT06614192) [18]. Importantly, the same phase 1 study also evaluated telisotuzumab adizutecan in patients with previously treated advanced gastric and gastroesophageal junction (GEJ) adenocarcinoma (*n* = 42), demonstrating an objective response rate of 29% and a clinical benefit rate of 71% [19]. A subsequent biomarker analysis confirmed that both MET protein overexpression and MET gene amplification were associated with enhanced antitumor activity in this cohort, and notably, 42% of responders lacked MET amplification, suggesting that protein expression rather than amplification status may be the more relevant predictive biomarker [20]. A Phase 2 study is now evaluating telisotuzumab adizutecan in combination with fluorouracil, leucovorin, and budigalimab in advanced gastric, GEJ, and esophageal adenocarcinoma [21]. Across these indications, patient selection has been based on varying proportions of MET IHC 3+ tumor cells— ≥ 50% in NSCLC, ≥ 10% in CRC—while optimal thresholds for upper GI adenocarcinomas have not yet been defined, underscoring the need for robust, standardized MET IHC assessment across tumor types.

Despite these encouraging developments, comprehensive data on the prevalence and clinical relevance of MET expression in adenocarcinomas of the upper GI tract remain limited, particularly regarding the distribution of MET expression across clinically relevant scoring thresholds in large, unselected cohorts. Prior studies have been constrained by small cohorts, heterogeneous scoring systems, or mixed histological subtypes, restricting translational insight and hindering clinical application. To address this gap, we conducted what is, to our knowledge, the largest real‐world cohort analysis of MET protein expression in upper GI adenocarcinomas to date, encompassing over 1500 patients of predominantly Caucasian descent. Using the clinically validated VENTANA MET (SP44) IHC assay, we systematically characterized the extent and distribution of MET expression across esophageal, EGJ, and gastric adenocarcinomas. Our study aims to define the landscape of MET protein overexpression in these malignancies and inform future biomarker‐driven therapeutic strategies, including the potential application of MET‐targeted ADCs.

## 2 Materials and Methods

### 2.1 Study Population and Tissue Samples

This retrospective cohort study included patients with histologically confirmed adenocarcinoma of the esophagus (EAC), esophagogastric junction, or stomach (GAC) who underwent surgical resection at the University Hospital Cologne. For EAC, patients were surgically treated with right transthoracic esophagectomy and a two‐field lymphadenectomy (mediastinal and abdominal lymph nodes). The intestinal passage was reconstructed with a high intrathoracic esophagogastrostomy as described previously [22]. Patients with GAC were treated with a subtotal distal or total gastrectomy with trans‐hiatal resection of the distal esophagus in case of a Siewert II‐tumor, followed by lymphadenectomy (level D2). A Roux‐en‐Y jejunal loop with gastrojejunostomy was the method for intestinal reconstruction. In EAC, neoadjuvant therapy consisted of either radiochemotherapy according to the Chemo‐radiotherapy for Oesophageal Cancer followed by Surgery Study (CROSS) protocol (carboplatin, paclitaxel and intensity‐modulated radiotherapy) or perioperative chemotherapy (Fluorouracil, Leucovorin, Oxaliplatin and Docetaxel: FLOT). For the treatment of patients with GAC, three different perioperative regimens were used in the last two decades: Cisplatin, 5‐Fluorouracil and Leucovorin (PFL), Medical Research Council Adjuvant Gastric Infusional Chemotherapy (Epirubicin, Cisplatin, Fluorouracil: MAGIC) and FLOT. Most patients were treated with MAGIC and FLOT protocols according to national German guidelines.

In the first 2 years after surgery, a clinical follow‐up was performed every 3 months, followed by annual check‐ups. The clinical follow‐up included a detailed check of the patient's medical history, physical examination, an ultrasound scan of the abdomen, a chest X‐ray, and, if required, additional diagnostics.

### 2.2 Tissue Microarray Construction

Tissue microarrays were generated using formalin‐fixed, paraffin‐embedded tumor specimens as reported previously [23]. Briefly, one tissue core with a diameter of 1.2 mm was transferred to the recipient paraffin block with a self‐constructed, semi‐automated precision instrument. Placenta tissue was included as a control.

### 2.3 MET Immunohistochemistry

MET protein expression was assessed by immunohistochemistry using the VENTANA MET (SP44) RxDx assay (Roche Diagnostics, Basel, Switzerland) on a VENTANA BenchMark staining platform according to the manufacturer's instructions. Appropriate positive and negative controls were included in each staining run. MET expression was scored by experienced pathologists blinded to clinical data. Membranous staining intensity was graded as 0 (no staining), 1+ (weak), 2+ (moderate), or 3+ (strong), and the percentage of tumor cells at each intensity level was recorded. The *H*‐score was calculated as: (1 x percentage of 1+ cells) + (2 x percentage of 2+ cells) + (3 x percentage of 3+ cells), yielding a score ranging from 0 to 300. For patient‐level analyses, the maximum *H*‐score and maximum 3+ percentage across multiple TMA cores per patient were used. Two stratification approaches were applied: (1) presence of any 3+ membranous staining (> 0% of tumor cells with 3+ intensity), with additional thresholds at ≥ 10% and ≥ 50% as used in clinical trials; and (2) *H*‐score ≥ 150, pre‐specified on two grounds: it is the minimum *H*‐score attainable by a tumor meeting the telisotuzumab vedotin NSCLC eligibility criterion of ≥ 50% tumor cells with 3+ staining intensity, and it reflects a biologically meaningful level of membranous overexpression. The robustness of this cutoff was assessed by sensitivity analyses across *H*‐score thresholds of 25, 50, 100, 150, 200, and 250 (Table S1), and by treating *H*‐score as a continuous variable (per 50‐point increase) in the multivariable Cox model.

### 2.4 MET Fluorescence In Situ Hybridization

MET fluorescence in situ hybridization (FISH) was performed as previously described [24]. Briefly, 4 μm sections from formalin‐fixed, paraffin‐embedded tissue blocks were hybridized overnight with the ZytoLight SPEC MET/CEN7 Dual Color Probe (ZytoVision, Bremerhaven, Germany). The amplification level was analyzed using a 100X objective and appropriate filter sets (DM5500 fluorescent microscope; Leica Biosystems, Wetzlar, Germany). Twenty tumor cell nuclei were evaluated by counting green (MET) and orange (centromere 7) signals. High amplification was defined as a MET/CEN7 ratio ≥ 2.0, an average MET gene copy number per cell ≥ 6.0, or ≥ 10% of the cells containing ≥ 15 MET signals. Intermediate amplification was defined as ≥ 50% of the cells containing ≥ 5 MET signals, provided none of the criteria for high‐level amplification were met [25]. On‐slide controls were conducted by screening normal epithelial tissue, fibroblasts, or lymphocytes.

### 2.5 Additional Biomarker Assessment

HER2 status was determined by FISH according to established gastroesophageal cancer scoring criteria. HER2 amplification was defined as HER2/CEN17 ratio ≥ 2.0, or a mean HER2 gene copy number ≥ 6.0 per cell in the presence of a negative ratio (< 2.0); methodological details have been described previously [26]. Claudin 18.2 expression was assessed by immunohistochemistry using the VENTANA CLDN18 (43‐14A) assay (Roche Diagnostics, Basel, Switzerland) and scored as positive if ≥ 75% of tumor cells exhibited moderate (2+) or strong (3+) membranous staining, as previously reported [27]. Mismatch repair (MMR) status was determined by immunohistochemistry as previously described [28]. Tumors with loss of MLH1 and PMS2 expression were classified as mismatch repair‐deficient (dMMR), while tumors with retained expression were classified as mismatch repair‐proficient (pMMR).

### 2.6 Clinical Data Collection

Clinical and pathological data were retrieved from electronic medical records and pathology reports, including age at diagnosis, sex, tumor location, TNM stage, treatment history (neoadjuvant chemotherapy or chemoradiotherapy), and survival outcomes.

### 2.7 Statistical Analysis

Descriptive statistics were used to summarize patient characteristics and MET expression distributions. Categorical variables were compared using Fisher's exact test or chi‐square test as appropriate. Continuous variables were compared using the Mann–Whitney U test or Kruskal‐Wallis test. The association between MET IHC status and FISH amplification was assessed using Fisher's exact test, and odds ratios with 95% confidence intervals were calculated.

Survival analysis was performed using the Kaplan–Meier method, with differences between groups assessed by the log‐rank test. Multivariate Cox proportional hazards regression was used to evaluate the independent prognostic significance of MET expression after adjusting for age, sex, T stage, N stage, and neoadjuvant therapy. Given the low prevalence of MET‐positive cases, penalized maximum likelihood estimation with L2 regularization (*λ* = 0.5) was employed to ensure model convergence and reduce small‐sample bias.

Concordance between MET expression in primary tumors and matched lymph node metastases was assessed using Cohen's kappa coefficient (*κ*), interpreted as: < 0 poor, 0–0.20 slight, 0.21–0.40 fair, 0.41–0.60 moderate, 0.61–0.80 substantial, and > 0.80 almost perfect agreement. Positive percent agreement (PPA) and negative percent agreement (NPA) were also calculated.

All statistical tests were two‐sided, and *p* < 0.05 were considered statistically significant. Analyses were performed using Python 3.12 with polars for data manipulation, SciPy for statistical tests, scikit‐survival for Kaplan–Meier estimation and log‐rank tests, and lifelines for Cox proportional hazards regression.

## 3 Results

### 3.1 Patient Characteristics

The study cohort comprised 1532 patients with upper GI adenocarcinoma (Table 1). Esophageal adenocarcinoma represented the majority of cases (*n* = 984, 64.2%), with gastric adenocarcinoma accounting for the remainder (*n* = 548, 35.8%). The cohort was predominantly male (75.7%) with a median age of 65 years (IQR 56–72). Most tumors presented at locally advanced stages, with T3 disease being most common (48.8%), followed by T2 (21.7%) and T1 (15.8%). Nodal involvement (N+) was present in 54.9% of patients (N1: 25.3%, N2: 15.5%, N3: 14.1%).

**TABLE 1:** Patient characteristics and association with MET expression status.

Characteristic	Total	< 150	≥ 150	p	Negative	Any 3+	p
Total N (patients)	1532 (100.0%)	1497 (97.7%)	35 (2.3%)		1508 (98.4%)	24 (1.6%)	
Tumor Entity, *n* (%)				0.48			0.14
Esophageal adenocarcinoma	984 (64.2%)	959 (64.1%)	25 (71.4%)		965 (64.0%)	19 (79.2%)	
Gastric adenocarcinoma	548 (35.8%)	538 (35.9%)	10 (28.6%)		543 (36.0%)	5 (20.8%)	
Sex, *n* (%)				0.82			1.00
Male	1160 (75.7%)	1133 (75.7%)	27 (77.1%)		1142 (75.7%)	18 (75.0%)	
Female	260 (17.0%)	255 (17.0%)	5 (14.3%)		256 (17.0%)	4 (16.7%)	
Unknown	112 (7.3%)	109 (7.3%)	3 (8.6%)		110 (7.3%)	2 (8.3%)	
Age, median (IQR)	65 (56–72)	65 (56–72)	66 (58–70)	0.91	65 (56–72)	66 (56–74)	0.90
T Stage, *n* (%)				0.04			0.06
T0	1 (0.1%)	1 (0.1%)	0 (0.0%)		1 (0.1%)	0 (0.0%)	
Tis	1 (0.1%)	1 (0.1%)	0 (0.0%)		1 (0.1%)	0 (0.0%)	
T1	242 (15.8%)	242 (16.2%)	0 (0.0%)		242 (16.0%)	0 (0.0%)	
T2	332 (21.7%)	328 (21.9%)	4 (11.4%)		329 (21.8%)	3 (12.5%)	
T3	748 (48.8%)	725 (48.4%)	23 (65.7%)		733 (48.6%)	15 (62.5%)	
T4	89 (5.8%)	85 (5.7%)	4 (11.4%)		85 (5.6%)	4 (16.7%)	
Unknown	119 (7.8%)	115 (7.7%)	4 (11.4%)		117 (7.8%)	2 (8.3%)	
N Stage, *n* (%)				0.57			0.65
N0	540 (35.2%)	531 (35.5%)	9 (25.7%)		534 (35.4%)	6 (25.0%)	
N1	388 (25.3%)	378 (25.3%)	10 (28.6%)		381 (25.3%)	7 (29.2%)	
N2	237 (15.5%)	231 (15.4%)	6 (17.1%)		233 (15.5%)	4 (16.7%)	
N3	216 (14.1%)	209 (14.0%)	7 (20.0%)		211 (14.0%)	5 (20.8%)	
Unknown	151 (9.9%)	148 (9.9%)	3 (8.6%)		149 (9.9%)	2 (8.3%)	
M Stage, *n* (%)				1.00			1.00
M0	16 (1.0%)	16 (1.1%)	0 (0.0%)		16 (1.1%)	0 (0.0%)	
M1	103 (6.7%)	99 (6.6%)	4 (11.4%)		101 (6.7%)	2 (8.3%)	
Unknown	1413 (92.2%)	1382 (92.3%)	31 (88.6%)		1391 (92.2%)	22 (91.7%)	
HER2 Status, *n* (%)				0.72			1.00
Positive	100 (6.5%)	99 (6.6%)	1 (2.9%)		99 (6.6%)	1 (4.2%)	
Negative	902 (58.9%)	881 (58.9%)	21 (60.0%)		888 (58.9%)	14 (58.3%)	
Not evaluable	530 (34.6%)	517 (34.5%)	13 (37.1%)		521 (34.5%)	9 (37.5%)	
Claudin 18.2 Status, *n* (%)				0.04			0.32
Positive	328 (21.4%)	325 (21.7%)	3 (8.6%)		325 (21.6%)	3 (12.5%)	
Negative	950 (62.0%)	922 (61.6%)	28 (80.0%)		932 (61.8%)	18 (75.0%)	
Not evaluable	254 (16.6%)	250 (16.7%)	4 (11.4%)		251 (16.6%)	3 (12.5%)	
MSI Status, *n* (%)				0.68			1.00
dMMR/MSI	70 (4.6%)	68 (4.5%)	2 (5.7%)		69 (4.6%)	1 (4.2%)	
pMMR/MSS	1267 (82.7%)	1238 (82.7%)	29 (82.9%)		1248 (82.8%)	19 (79.2%)	
Not evaluable	195 (12.7%)	191 (12.8%)	4 (11.4%)		191 (12.7%)	4 (16.7%)	

MET expression was evaluated using two complementary stratification approaches. First, given that regulatory approvals for MET‐targeted ADCs have been based on the percentage of tumor cells exhibiting 3+ membranous staining intensity, we stratified patients by the presence of any 3+ staining. Second, recognizing that 3+ intensity represents only one component of the composite *H*‐score, we also evaluated the full *H*‐score distribution. We pre‐specified an H‐score threshold of ≥ 150 because it represents the minimum *H*‐score attainable under the NSCLC‐trial eligibility pattern of ≥ 50% tumor cells with 3+ staining intensity and captures biologically meaningful membranous overexpression. Sensitivity analyses across *H*‐score thresholds of 25–250 confirmed that the adjusted hazard ratio for overall survival increased monotonically with higher cutoffs and plateaued between ≥ 100 and ≥ 200 (adjusted HR 1.69–2.10, all *p* < 0.05), indicating that ≥ 150 sits within a robust range rather than at an arbitrary inflection point (Table S1). *H*‐score modeled as a continuous variable was independently prognostic in the same multivariable Cox model (HR 1.14 per 50‐point increase, 95% CI 1.04–1.25, *p* = 0.005), confirming that the association with outcome is not threshold‐dependent.

Using the *H*‐score ≥ 150 threshold, 35 patients (2.3%) demonstrated high MET expression. When applying the any 3+ criterion, 24 patients (1.6%) were classified as MET‐positive. Patients with high MET expression (*H*‐score ≥ 150) showed a significant association with advanced T stage (*p* = 0.04), with 65.7% presenting with T3 disease and 11.4% with T4 disease, compared to 48.4% and 5.7%, respectively, in the low‐expression group. Notably, no T1 tumor exhibited 3+ membranous staining, although lower‐level MET expression was detected at the expected frequency in this subgroup (*H*‐score > 0 in 21/242 [8.7%], up to a maximum *H*‐score of 90), indicating that the TMA cores were informative for MET.

MET expression status showed no significant association with tumor entity (*p* = 0.48), sex (*p* = 0.82), age (*p* = 0.91), nodal stage (*p* = 0.57), distant metastasis (*p* = 1.00), HER2 status (*p* = 0.72), or microsatellite instability (MSI) status (*p* = 1.00). Similar patterns were observed when stratifying by any 3+ MET expression, though the association with T stage showed a trend toward significance (*p* = 0.06) rather than reaching statistical significance.

### 3.2 MET Expression Distribution

**FIGURE 1:**
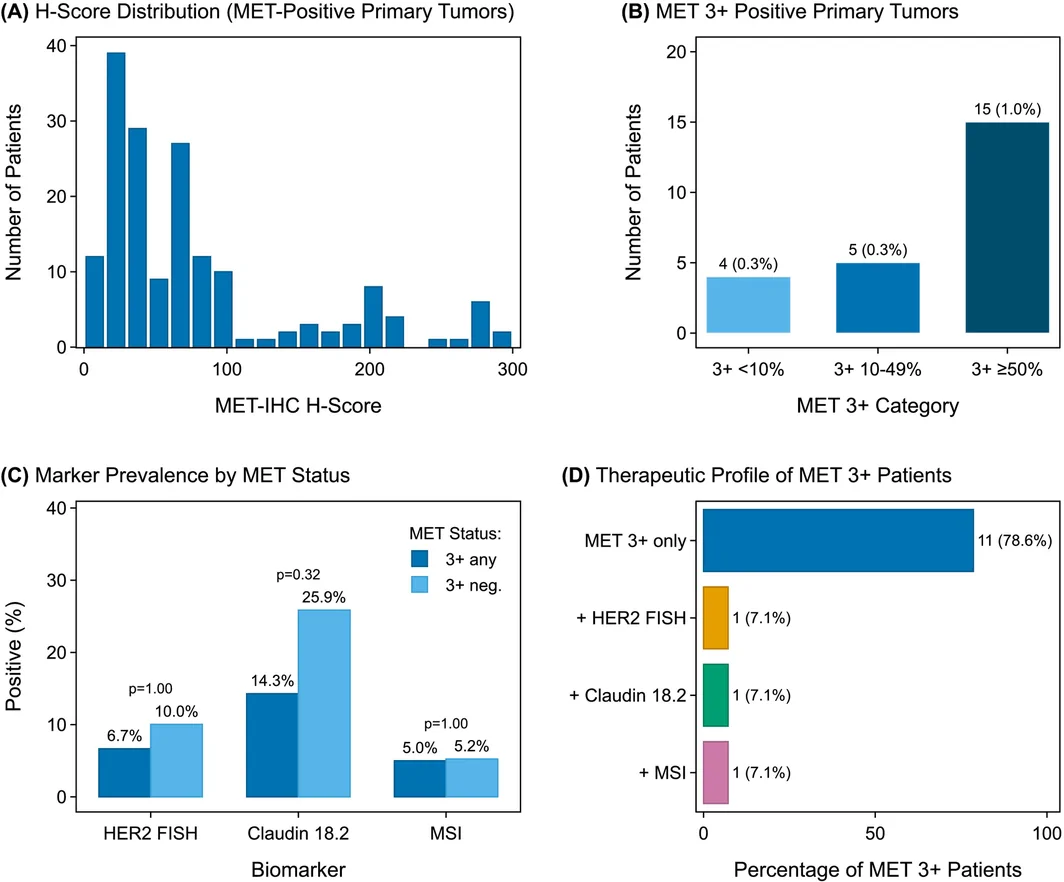
MET Expression Distribution and Association with Druggable Alterations. Distribution of MET expression and its relationship to other clinically actionable biomarkers in esophageal and gastric adenocarcinoma (patient‐level analysis, primary tumors only). (A) *H*‐score distribution among MET‐positive patients (*H*‐score > 0; maximum per patient if multiple tissue microarray spots). (B) Distribution of 3+ positive patients by percentage of tumor cells with 3+ membranous staining intensity as a proportion of the full primary‐tumor cohort, stratified by clinically relevant thresholds (< 10%, 10%–49%, ≥ 50%). (C) Prevalence of HER2 FISH positivity, positive Claudin 18.2 expression status, and microsatellite instability (MSI) in patients stratified by MET 3+ status (3+ any vs. 3+ negative). *p* values from Fisher's exact test. (D) Therapeutic profile among MET 3+ patients with evaluable HER2, Claudin 18.2, and MSI status showing the proportion with no additional druggable alterations (MET 3+ only) versus those with concurrent HER2 FISH positivity, Claudin 18.2 expression, or MSI.

The distribution of MET protein expression across the cohort is illustrated in Figure 1A,B. Among patients with detectable MET expression, *H*‐scores demonstrated a right‐skewed distribution with the majority of cases exhibiting low‐to‐moderate expression levels (Figure 1A). The modal *H*‐score range was 25–50, with progressively fewer cases observed at higher expression levels.

When categorizing patients by percentage of tumor cells with 3+ membranous staining intensity—the metric used for patient selection in MET‐targeted ADC trials—we identified 24 patients (1.6%) with any 3+ staining (Figure 1B). Of these, 4 patients (0.3%) demonstrated 3+ staining in < 10% of tumor cells, 5 patients (0.3%) exhibited 3+ staining in 10%–49% of cells, and 15 patients (1.0%) showed 3+ staining in ≥ 50% of tumor cells. The latter group would meet the eligibility threshold established for telisotuzumab vedotin in NSCLC (≥ 50% 3+ staining), while the combined group with ≥ 10% 3+ staining (*n* = 20, 1.3%) would qualify under the criteria applied in colorectal cancer trials.

### 3.3 Correlation Between MET Protein Expression and Gene Amplification

To evaluate the association between MET protein expression and gene amplification, we analyzed 817 patients from the esophageal adenocarcinoma cohort for which paired fluorescence in situ hybridization (FISH) data were available. MET‐IHC was strongly associated with FISH amplification status across all evaluated thresholds (Table 2). Using the *H*‐Score ≥ 150 threshold, 63.6% (14/22) of IHC‐positive patients showed FISH amplification compared to 2.4% (19/795) of IHC‐negative patients (OR 71.5, *p* < 0.001). The 3+ staining percentage approach demonstrated an even stronger association: among patients with any 3+ staining, 75.0% (12/16) were FISH amplified versus 2.6% (21/801) of 3+ negative patients (OR 111.4, *p* < 0.001). Stricter 3+ thresholds further enriched for FISH amplification, with ≥ 50% 3+ staining identifying patients with an 80.0% (8/10) FISH amplification rate (OR 125.1, *p* < 0.001).

**TABLE 2:** Association between MET‐IHC and MET FISH amplification status in esophageal adenocarcinoma (*n* = 817 patients with paired data).

MET‐IHC threshold	*n* (IHC+)	FISH Amp in IHC+	FISH Amp in IHC−	Odds Ratio	*p*
H‐Score ≥ 150	22	14/22 (63.6%)	19/795 (2.4%)	71.5	< 0.001
Any 3+	16	12/16 (75.0%)	21/801 (2.6%)	111.4	< 0.001
≥ 10% 3+	13	10/13 (76.9%)	23/804 (2.9%)	113.2	< 0.001
≥ 50% 3+	10	8/10 (80.0%)	25/807 (3.1%)	125.1	< 0.001

*Note:* Patient‐level analysis using maximum *H*‐score and 3+ percentage across primary tumor TMA spots per patient. Two IHC stratification approaches are compared: *H*‐score threshold (≥ 150) and 3+ staining percentage. FISH amplification rates and odds ratios are shown for IHC‐positive versus IHC‐negative groups. Fisher's exact test was used for all comparisons.

### 3.4 Association of MET Expression With Druggable Alterations

To assess the relationship between MET expression and other clinically actionable biomarkers, we analyzed the prevalence of HER2 FISH positivity, Claudin 18.2 expression, and MSI in patients stratified by MET 3+ status (Figure 1C,D). Among 1002 patients with evaluable HER2 status, those with any MET 3+ staining (*n* = 15) showed comparable rates of HER2 FISH positivity to MET 3+ negative patients (*n* = 987): 6.7% versus 10.0% (*p* = 1.00). Among 1278 patients evaluable for Claudin 18.2, positivity showed a trend toward lower prevalence in MET 3+ patients (*n* = 21) compared to MET 3+ negative patients (*n* = 1257): 14.3% versus 25.9% (*p* = 0.32), consistent with the significant inverse association observed in Table 1. Among 1337 patients evaluable for MSI status, comparable rates were observed between MET 3+ patients (*n* = 20) and MET 3+ negative patients (*n* = 1317): 5.0% versus 5.2% (*p* = 1.00).

Among the 12 MET 3+ patients with evaluable status for all three markers, the majority (*n* = 8, 66.7%) exhibited no additional druggable alterations, representing a patient population for whom MET‐targeted ADC therapy may be the primary targeted treatment option. The remaining patients showed overlap with HER2 FISH positivity (*n* = 1, 8.3%), Claudin 18.2 expression (*n* = 2, 16.7%), or MSI (*n* = 1, 8.3%), indicating potential alternative or combination therapeutic strategies. Analogous analysis using the *H*‐Score ≥ 150 threshold (Figure S1) showed similar patterns, with the inverse association between MET expression and Claudin 18.2 positivity reaching statistical significance (*p* = 0.04).

### 3.5 Survival Analysis

**FIGURE 2:**
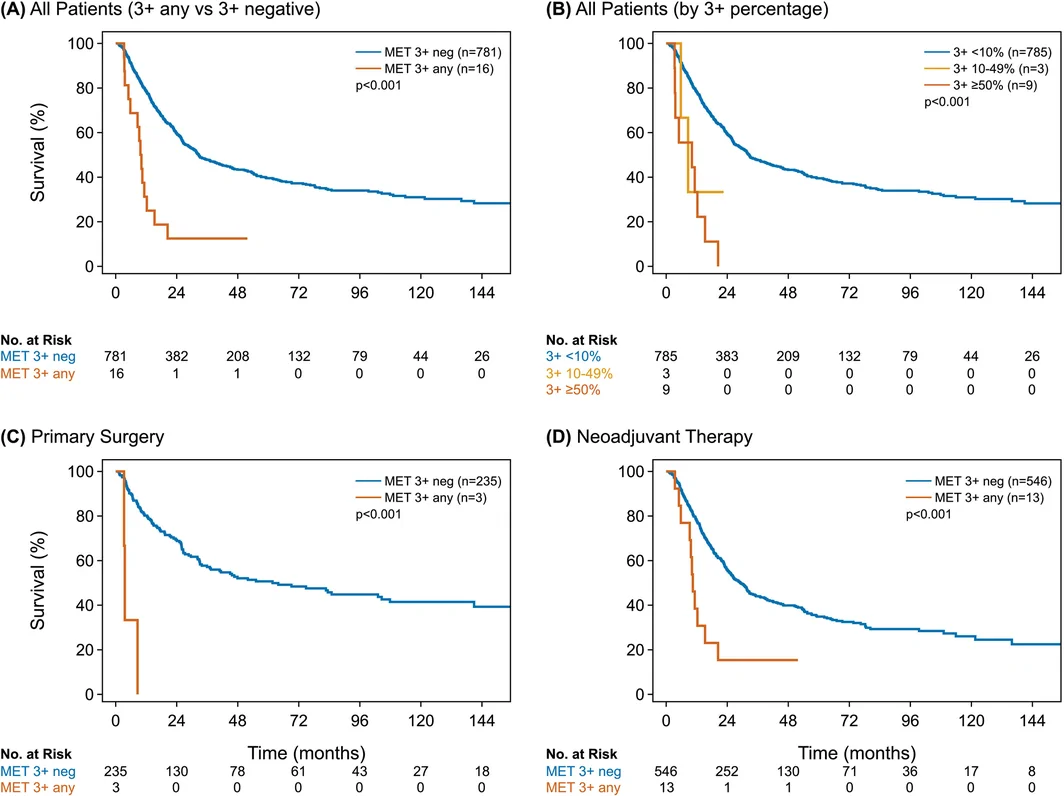
Survival Analysis by MET 3+ Status. Kaplan–Meier curves showing overall survival in esophageal adenocarcinoma stratified by MET IHC 3+ expression (patient‐level analysis, primary tumors only). (A) All patients comparing MET 3+ any versus MET 3+ negative. (B) All patients stratified by percentage of tumor cells with 3+ membranous staining intensity (< 10%, 10%–49%, ≥ 50%). (C) Patients treated with primary surgery without neoadjuvant therapy. (D) Patients who received neoadjuvant therapy prior to surgery. Numbers at risk are shown below each panel. Log‐rank test *p* values are displayed within each panel.

To evaluate the prognostic significance of MET expression, we performed Kaplan–Meier survival analysis in the esophageal adenocarcinoma cohort (*n* = 797 patients with survival data; Figure 2). Patients with any 3+ MET staining (*n* = 16) demonstrated significantly worse overall survival compared to MET 3+ negative patients (*n* = 781; *p* < 0.001, log‐rank test; Figure 2A). Median overall survival was 9.7 months in MET 3+ patients compared to 32.7 months in MET 3+ negative patients. The 5‐year survival rate was 12.5% for MET 3+ patients versus 39.6% for MET 3+ negative patients. When stratifying by the percentage of tumor cells with 3+ staining intensity, we observed a significant association between higher 3+ percentage and poorer survival across the three groups (< 10%, *n* = 785; 10%–49%, *n* = 3; ≥ 50%, *n* = 9; *p* < 0.001; Figure 2B).

Subgroup analysis by treatment approach revealed consistent findings. In patients who underwent primary surgery without neoadjuvant therapy (Figure 2C), MET 3+ status (*n* = 3) was significantly associated with worse overall survival compared to MET 3+ negative patients (*n* = 235; *p* < 0.001). Similarly, in patients who received neoadjuvant chemotherapy or chemoradiotherapy prior to surgery (Figure 2D), MET 3+ expression (*n* = 13) was associated with significantly poorer prognosis compared to MET 3+ negative patients (*n* = 546; *p* < 0.001). Prevalence of MET 3+ staining did not differ significantly between treatment‐naïve and neoadjuvant‐treated patients (1.0% vs. 2.3%, Fisher's exact *p* = 0.20), nor did the underlying *H*‐score or 3+ percentage distributions (Mann–Whitney *p* = 0.72 and *p* = 0.16).

Analogous survival analysis using the *H*‐Score ≥ 150 threshold (Figure S2) demonstrated similar prognostic significance, confirming that MET overexpression is associated with poor outcomes regardless of the stratification approach employed. Notably, stratification by *H*‐Score range (< 50, 50–149, ≥ 150) revealed clearer separation of survival curves compared to the 3+ percentage approach, suggesting that the continuous *H*‐Score may provide superior prognostic discrimination in this cohort. Analogous survival analysis in the gastric adenocarcinoma cohort demonstrated similar prognostic associations (Figures S3 and S4).

Given the association of MET 3+ expression with advanced T stage (Table 1), we sought to determine whether the prognostic effect of MET expression was independent of established clinicopathological factors. Multivariate Cox proportional hazards regression in the esophageal adenocarcinoma cohort (Table 3) confirmed this association. After adjusting for age, sex, tumor stage (T and N), and neoadjuvant therapy, MET 3+ positivity remained independently associated with worse overall survival (HR = 2.22, 95% CI 1.29–3.83, *p* = 0.004). Advanced T‐stage (T3‐4 vs. T1‐2) was also independently associated with mortality (HR = 1.40, 95% CI 1.20–1.64, *p* < 0.001), as was increasing age (HR = 1.10 per 10 years, 95% CI 1.02–1.18, *p* = 0.009). Sex, nodal stage, and neoadjuvant therapy status were not significant independent predictors in this model. Multivariate Cox regression using the *H*‐Score ≥ 150 threshold (Table S2) yielded concordant results, demonstrating that MET overexpression remains an independent prognostic factor regardless of the stratification method used.

**TABLE 3:** Multivariate Cox proportional hazards regression for overall survival in esophageal adenocarcinoma (*n* = 667 patients, 382 events).

Variable	Category	HR	95% CI	*p*
MET 3+	Any versus None	2.22	1.29–3.83	0.004
Age	Per 10 years	1.10	1.02–1.18	0.009
Sex	Male versus Female	1.21	0.96–1.54	0.11
T‐stage	T3‐4 versus T1‐2	1.40	1.20–1.64	< 0.001
N‐stage	*N*+ versus N0	1.71	0.74–3.93	0.21
Neoadjuvant therapy	Yes versus No	1.13	0.96–1.33	0.13

*Note:* HR > 1 indicates increased risk of death. Reference categories: MET 3+ negative, female sex, T‐stage T1‐2, N‐stage N0, no neoadjuvant therapy. Penalized maximum likelihood estimation (L2, *λ* = 0.5) was used to ensure model convergence given the sparse MET 3+ positive cases (*n* = 13).

Abbreviations: CI, confidence interval; HR, hazard ratio.

### 3.6 MET Expression Concordance Between Primary Tumor and Lymph Node Metastasis

To assess the consistency of MET expression between primary tumors and matched lymph node metastases, we analyzed 359 patients from the combined esophageal and gastric adenocarcinoma cohort who had evaluable MET IHC data from both sites (Table 4). MET 3+ positivity was rare at both sites: 4 patients (1.1%) showed any 3+ staining in the primary tumor and 5 patients (1.4%) in the lymph node metastasis. Of the 4 primary‐positive cases, only 2 (50%) were also positive in the lymph node metastasis (positive percent agreement, PPA = 50.0%). The remaining 2 primary‐positive cases showed loss of MET 3+ expression in the metastasis, while 3 cases showed de novo MET 3+ expression in the lymph node metastasis that was absent in the primary tumor.

**TABLE 4:** Concordance of MET 3+ staining status between matched primary tumor and lymph node metastasis samples from the combined esophageal and gastric adenocarcinoma cohort.

Statistic	MET 3+ any	MET 3+ ≥ 10%	MET 3+ ≥ 50%
Paired cases (*n*)	359	359	359
Primary tumor positive	4 (1.1%)	4 (1.1%)	2 (0.6%)
Lymph node metastasis positive	5 (1.4%)	5 (1.4%)	3 (0.8%)
Both positive	2 (0.6%)	2 (0.6%)	0 (0.0%)
Both negative	352 (98.1%)	352 (98.1%)	354 (98.6%)
Primary+, LN−	2 (0.6%)	2 (0.6%)	2 (0.6%)
Primary−, LN+	3 (0.8%)	3 (0.8%)	3 (0.8%)
Cohen's *κ*	0.44 (moderate)	0.44 (moderate)	−0.01 (poor)
PPA	50.0%	50.0%	0.0%
NPA	99.2%	99.2%	99.2%

*Note:* Three clinically relevant thresholds are compared: Any 3+ staining (> 0% of tumor cells), ≥ 10% of cells with 3+ staining (eligibility threshold used in colorectal cancer trials), and ≥ 50% of cells with 3+ staining (eligibility threshold used in NSCLC trials). Cohen's *κ* measures agreement beyond chance (interpretation: < 0 = poor, 0–0.20 = slight, 0.21–0.40 = fair, 0.41–0.60 = moderate, 0.61–0.80 = substantial, > 0.80 = almost perfect). Analysis uses maximum 3+ percentage when multiple TMA spots were available per tissue type per patient.

Abbreviations: NPA, negative percent agreement (% of primary‐negative cases also negative in lymph node metastasis); PPA, positive percent agreement (% of primary‐positive cases also positive in lymph node metastasis).

Cohen's kappa (*κ*), which accounts for chance agreement in the setting of low prevalence, was 0.44 for the any 3+ threshold, indicating only moderate agreement. Similar findings were observed at the ≥ 10% threshold (*κ* = 0.44, PPA = 50.0%). At the ≥ 50% threshold, concordance was poor (*κ* = −0.01) with no primary‐positive cases showing concordant positivity in the lymph node metastasis (PPA = 0.0%). These findings indicate substantial heterogeneity in MET expression between primary tumors and lymph node metastases, suggesting that assessment of both sites may be warranted when evaluating patients for MET‐targeted therapy. Analogous concordance analysis using the *H*‐Score ≥ 150 threshold (Table S3) showed similar patterns of heterogeneity.

## 4 Discussion

In this large real‐world cohort of more than 1500 patients with adenocarcinomas of the upper GI tract, we provide a comprehensive characterization of MET protein expression using a clinically validated immunohistochemical assay. Several key findings emerge from this analysis. First, high‐level MET protein overexpression is a rare but clearly identifiable event in esophageal, esophagogastric junction, and gastric adenocarcinomas. Second, MET overexpression is strongly associated with MET gene amplification, advanced local tumor stage, and markedly poor survival outcomes. Third, MET‐positive tumors frequently lack other established druggable alterations, underscoring MET as a potentially exclusive therapeutic target in this subset. Finally, substantial discordance between primary tumors and matched lymph node metastases highlights pronounced spatial heterogeneity of MET expression.

The overall prevalence of MET overexpression observed in our cohort was low, irrespective of the stratification approach applied. Depending on the definition used, 1.6% of patients exhibited any MET IHC 3+ staining, 1.3% met the ≥ 10% 3+ threshold applied in colorectal cancer trials, and 1.0% fulfilled the ≥ 50% 3+ criterion used for patient selection in NSCLC studies of telisotuzumab vedotin. Using an *H*‐score–based definition, 2.3% of tumors demonstrated high MET expression (*H*‐score ≥ 150). These frequencies are lower than those reported in some earlier, smaller studies, which often relied on heterogeneous antibodies, non‐standardized scoring systems, or enrichment for advanced disease. The use of the FDA‐approved VENTANA MET (SP44) assay and patient‐level maximum scoring across multiple tissue cores strengthens the robustness and clinical translatability of our estimates.

Our findings are consistent with prior literature demonstrating that high‐level MET overexpression represents a rare but biologically aggressive event in gastric and esophagogastric adenocarcinomas. In localized disease, Janjigian et al. showed that MET protein expression was common but true gene amplification was absent, suggesting that MET‐driven biology is uncommon in early‐stage tumors [29]. In contrast, studies enriched for advanced disease have consistently linked both MET overexpression and amplification to poor survival outcomes [30, 31, 32, 33, 34]. Several large series have further established that MET IHC 3+ expression is strongly concordant with gene amplification, though overexpression can also occur independently of copy number gains [32, 33, 35]. Using a modified scoring system, Zhang et al. reported a 6% IHC 3+ rate with 94.6% IHC–amplification concordance in 816 Chinese patients [35], while Lennerz et al. identified MET amplification as the most aggressive molecular subgroup in esophagogastric adenocarcinomas and provided early evidence of crizotinib responsiveness [34]. The heterogeneity in reported prevalence rates across studies largely reflects differences in antibodies, scoring systems, disease stage, and cohort ethnicity, reinforcing the need for standardized MET IHC assessment using clinically validated assays.

Despite its rarity, MET overexpression clearly delineates a biologically aggressive tumor subset. High MET expression was significantly associated with advanced T stage, and no T1 tumors demonstrated MET overexpression, suggesting that MET activation may preferentially occur during later phases of tumor progression. Consistent with this notion, MET overexpression conferred a markedly adverse prognosis. Patients with any MET IHC 3+ staining experienced a more than threefold reduction in median overall survival and a substantially lower 5‐year survival rate compared with MET‐negative patients. Importantly, this effect remained statistically significant after adjustment for established clinicopathological prognostic factors, confirming MET overexpression as an independent marker of poor outcome. These findings are consistent with preclinical data implicating MET signaling in promoting invasion, epithelial–mesenchymal transition, and metastatic dissemination, and they extend prior observations by demonstrating robust prognostic relevance in a large, uniformly analyzed cohort. Consistent with MET overexpression marking a biologically distinct entity, proteomic analysis of MET‐amplified esophageal adenocarcinomas from our institution identified a specific molecular phenotype enriched for alternative RNA splicing and androgen receptor signaling proteins, suggesting additional vulnerable pathways beyond MET itself that may inform combination treatment strategies [24].

A particularly important observation is the strong correlation between MET protein overexpression and MET gene amplification. However, not all MET‐overexpressing tumors were amplified, indicating that protein overexpression cannot be fully explained by gene copy number gains alone. This finding is clinically relevant, as emerging data from ADC trials indicate that the therapeutic efficacy of MET‐targeted ADCs is not primarily driven by MET gene amplification per se, but rather by high‐level MET protein expression at the tumor cell surface. In pivotal and exploratory studies of telisotuzumab vedotin and telisotuzumab adizutecan, patient selection was explicitly based on the proportion of tumor cells exhibiting 3+ membranous MET staining, with different quantitative thresholds applied across tumor entities. In NSCLC, a stringent cutoff of ≥ 50% MET 3+ tumor cells was required to achieve clinically meaningful response rates [16, 36], whereas in metastatic colorectal cancer, lower thresholds (≥ 10% MET 3+ cells) were sufficient to identify responsive tumors [18]. This principle extends to upper GI adenocarcinomas: biomarker data from the gastric/GEJ adenocarcinoma cohort of the telisotuzumab adizutecan phase 1 study demonstrated a negative correlation between c‐Met protein expression level and tumor size change (R = −0.59), and notably, 42% of objective responders lacked MET gene amplification [20]. Importantly, this biomarker analysis employed the same SP44 antibody clone used in our study, ensuring direct comparability of MET expression assessments across datasets. Collectively, these findings underscore that MET protein expression assessed by IHC—rather than amplification status alone—may be the more clinically actionable predictive biomarker for ADC‐based therapies.

These observations suggest that the presence of a critical fraction of tumor cells with very high surface MET density—rather than uniform overexpression across the entire tumor mass—is a key determinant of ADC internalization, payload delivery, and antitumor activity. The enrichment of MET‐amplified tumors in the GEA trial population (29% vs. approximately 3% in real‐world data) [20] further highlights that current clinical trials are enriched for MET‐driven tumors and may not reflect the true prevalence of actionable MET expression in unselected patient populations. Against this background, our study is the first to systematically apply these clinically established MET 3+ scoring concepts to a large, unselected cohort of adenocarcinomas of the upper GI tract, providing the real‐world prevalence data needed to contextualize trial‐derived efficacy estimates.

As the optimal cutoff of MET expression for predicting therapeutic efficacy of MET‐directed ADCs in adenocarcinomas of the upper GI tract has not yet been defined, it is biologically and clinically justified to apply immunohistochemical scoring systems that more comprehensively reflect both the intensity and the spatial extent of MET expression in tumor cells. We therefore additionally employed the internationally established histoscore (*H*‐score), which integrates these parameters into a continuous measure of protein overexpression. In our cohort, the *H*‐score provided superior prognostic discrimination of relevant MET‐associated phenotypes and robustly stratified patients with adverse outcomes, supporting its evaluation as a candidate patient stratification biomarker in future prospective trials of MET‐directed ADCs. Whether *H*‐score–based stratification also predicts response to MET‐targeted therapy is a hypothesis that our observational data cannot test and that warrants dedicated prospective investigation.

The therapeutic context of MET overexpression is further informed by its relationship with other actionable biomarkers. MET‐positive tumors rarely overlapped with HER2 amplification or MSI, and showed a significantly lower prevalence of Claudin 18.2 expression. Notably, two‐thirds of MET 3+ tumors lacked any additional established druggable alteration. This observation defines a small but clinically important patient population for whom MET‐targeted ADCs could represent the primary biomarker‐driven treatment option. Given the limited efficacy of standard systemic therapies in this molecular subset, and the encouraging early‐phase activity of telisotuzumab adizutecan in advanced gastric/GEJ adenocarcinoma (ORR 29%, CBR 71%) [19], these findings provide a strong rationale for future prospective evaluation of MET‐directed ADCs in upper GI adenocarcinomas. To test whether this prognostic effect is intrinsic to MET biology or reflects the relative absence of alternative biomarker‐directed therapies in this subgroup, we performed a pre‐specified subgroup analysis restricted to esophageal patients without HER2 amplification, Claudin 18.2 expression, or dMMR/MSI and with complete covariate data (*n* = 275, 175 events; Table S4). Within this subgroup, MET 3+ patients had substantially shorter overall survival (median 9.3 vs. 25.5 months, log‐rank *p* < 0.001), and the multivariable‐adjusted hazard ratio (HR 4.15, 95% CI 2.05–8.38) was numerically larger than in the full cohort (Table 3, HR 2.22), arguing against differential treatment access as the principal driver.

Our analysis also reveals pronounced spatial heterogeneity of MET expression. Concordance between primary tumors and matched lymph node metastases was only moderate at best and poor at higher 3+ thresholds, with both loss and gain of MET overexpression observed in metastatic sites. These findings have important implications for clinical practice and trial design. Reliance on a single tissue specimen—particularly archival primary tumor tissue—may lead to misclassification of MET status. Whenever feasible, assessment of metastatic tissue or multi‐site sampling may be warranted to optimize patient selection for MET‐targeted therapies.

A substantial proportion of patients received neoadjuvant therapy, raising the possibility that treatment exposure biased the MET–survival association. Three observations argue against this: multivariable Cox regression adjusted for neoadjuvant therapy (Table 3), the MET 3+ effect was of comparable magnitude in both treatment subgroups (Figure 2C,D), and MET prevalence and distribution did not differ between arms. We cannot, however, formally exclude that cytotoxic or radiation exposure modulates MET expression, since IHC in neoadjuvant‐treated patients was assessed on post‐treatment residual tumor; paired pre‐ and post‐treatment biopsies will be needed to resolve this in future prospective MET‐ADC trials.

Several limitations should be acknowledged. The retrospective, single‐center design introduces potential selection biases. Although our cohort is one of the largest reported to date, the absolute number of MET‐positive cases remains small, reflecting the biological rarity of this alteration and limiting statistical power for subgroup analyses. Importantly, our study was designed to characterize prevalence and prognostic significance and is not a predictive biomarker validation; whether MET immunohistochemistry (at any threshold) predicts response to MET‐directed ADCs in upper GI adenocarcinomas must be evaluated in prospective therapeutic trials. The TMA‐based design is a further limitation: most tumors were represented by a single core, which cannot fully capture intratumoral heterogeneity, and rare MET 3+ foci in early‐stage tumors could therefore have been missed. However, the detection of lower‐level MET staining in a subset of T1 tumors suggests the cores were biologically informative, and the complete absence of high‐level MET overexpression in 242 T1 cases is consistent with the known rarity of MET amplification (the molecular event most strongly linked to the 3+ IHC phenotype) in non‐metastatic upper GI adenocarcinoma. Table 1 also contains substantial missing data. Clinical staging variables drawn from the retrospective record were missing in < 10% of cases (sex 7.3%, T 7.8%, N 9.9%), whereas M‐stage was undocumented in 92.2%, reflecting the curative‐intent resectional nature of the cohort; M‐stage was not used as a Cox covariate. Ancillary biomarkers (HER2, Claudin 18.2, MSI) were not evaluable in 12.7%–34.6% of cases, predominantly due to TMA cores with insufficient tumor or lost during sectioning. All analyses were complete‐case with per‐variable denominators, and the consistency of the MET–survival association across stratifications and subgroups argues against major bias, but informative missingness cannot be formally excluded.

In conclusion, this study provides the most comprehensive real‐world prevalence and prognostic characterization of MET protein expression in upper GI adenocarcinomas to date. MET overexpression defines a rare but highly aggressive tumor subset, confers independent adverse prognostic significance, and frequently occurs in the absence of other actionable biomarkers. Together with early‐phase clinical data demonstrating activity of the MET‐directed ADC telisotuzumab adizutecan in advanced gastric/GEJ adenocarcinoma, these observations position MET‐directed ADCs as a biologically rational therapeutic hypothesis for this subset. The prevalence and distribution data reported here are intended to inform the design and patient‐selection criteria of ongoing and future prospective trials. Whether MET IHC—using the current 3+ percentage thresholds, the *H*‐score, or a combined metric—functions as a predictive biomarker for response to MET‐directed ADCs is a question that can only be answered in prospective therapeutic studies.

## Author Contributions

**Tillmann Bedau:** conceptualization, investigation, writing – original draft, methodology, visualization, writing – review and editing, software, formal analysis, data curation, project administration. **Thomas Zander:** resources, supervision, writing – review and editing. **Hans Anton Schlößer:** writing – review and editing, supervision, resources. **Hakan Alakus:** writing – review and editing, resources, supervision, writing – original draft, methodology. **Onur Mustafov:** methodology, writing – review and editing, writing – original draft, investigation, visualization, formal analysis, data curation. **Reinhard Büttner:** supervision, resources, writing – review and editing. **Christiane Bruns:** writing – review and editing, resources, supervision. **Alexander Quaas:** conceptualization, investigation, funding acquisition, writing – original draft, writing – review and editing, methodology, validation, project administration, data curation, supervision, resources.

## Ethics Statement

The study was approved by the Ethics Committee of the University Hospital Cologne (protocol number 21–1146). Written informed consent was waived due to the retrospective nature of the study.

## Conflicts of Interest

The authors declare no conflicts of interest.

## Supporting information


**Figure S1:** Association of MET expression with druggable alterations (*H*‐Score ≥ 150 Stratification). Relationship between MET protein expression and other clinically actionable biomarkers in combined esophageal and gastric adenocarcinoma using the *H*‐Score ≥ 150 threshold (patient level analysis, primary tumors only). (A) Prevalence of HER2 FISH positivity, Claudin 18.2 expression, and microsatellite instability (MSI) in patients stratified by MET *H*‐Score (≥ 150 vs. < 150). *p* values from Fisher's exact test. (B) Therapeutic profile of MET *H*‐Score ≥ 150 patients showing the proportion with no additional druggable alterations (MET ≥ 150 only) versus those with concurrent HER2 FISH positivity, Claudin 18.2 expression, or MSI.
**Figure S2:** Survival analysis by MET H‐Score ≥ 150 status. Kaplan–Meier curves showing overall survival in esophageal adenocarcinoma stratified by MET H‐Score threshold (patient‐level analysis, primary tumors only). (A) All patients comparing H‐Score ≥ 150 versus H‐Score ≥ 150 versus H‐Score < 150 (B) All patients stratified by H‐Score range (< 50, 50–149, ≥ 150). (C) Patients treated with primary surgery without neoadjuvant therapy. (D) Patients who received neoadjuvant therapy prior to surgery. Numbers at risk are shown below each panel. Log‐rank test *p* values are displayed within each panel.
**Figure S3:** Survival analysis by MET 3+ status. Kaplan–Meier curves showing overall survival in gastric adenocarcinoma stratified by MET IHC 3+ expression (patient‐level analysis, primary tumors only). (A) All patients comparing MET 3+ any versus MET 3+ negative. (B) All patients stratified by percentage of tumor cells with 3+ membranous staining intensity (< 10%, 10%–49%, ≥ 50%). (C) Patients treated with primary surgery without neoadjuvant therapy. (D) Patients who received neoadjuvant therapy prior to surgery. Log‐rank test *p* values are displayed within each panel.
**Figure S4:** Survival analysis by MET H‐Score ≥ 150 status. Kaplan–Meier curves showing overall survival in gastric adenocarcinoma stratified by MET H‐Score threshold (patient‐level analysis, primary tumors only). (A) All patients comparing H‐Score ≥ 150 versus H‐Score ≥ 150 versus H‐Score < 150. (B) All patients stratified by H‐Score range (< 50, 50–149, ≥ 150). (C) Patients treated with primary surgery without neoadjuvant therapy. (D) Patients who received neoadjuvant therapy prior to surgery Log‐rank test *p* values are displayed within each panel.
**Table S1:** Sensitivity analysis of H‐score thresholds in the esophageal adenocarcinoma cohort. Prevalence is calculated among 984 primary‐tumor patients with evaluable MET IHC (patient‐level analysis, maximum H‐score per patient across tissue microarray spots). MET IHC–FISH concordance is calculated among 817 patients with paired IHC and FISH data. Univariable log‐rank tests were performed on the esophageal adenocarcinoma cohort with available survival data. Multivariable Cox proportional hazards regression (*n* = 667 patients, 382 events) adjusted for age (per 10 years), sex, T‐stage (T3–4 vs. T1–2), N‐stage (N+ vs. N0), and neoadjuvant therapy, with L2 penalization (λ = 0.5). The bold row indicates the primary cutoff used throughout the manuscript. The monotonic increase in odds ratios for FISH amplification and adjusted hazard ratios for overall survival across cutoffs demonstrates the robustness of the pre‐specified H‐score ≥ 150 threshold. HR, hazard ratio; CI, confidence interval.
**Table S2:** Multivariate Cox proportional hazards regression for overall survival in esophageal adenocarcinoma using the H‐Score ≥ 150 threshold (*n* = 667 patients, 382 events). HR, hazard ratio; CI, confidence interval. HR > 1 indicates increased risk of death. Reference categories: MET H Score < 150, female sex, T‐stage T1‐2, N‐stage N0, no neoadjuvant therapy. Penalized maximum likelihood estimation (L2, λ = 0.5) was used to ensure model convergence given the sparse MET H‐Score ≥ 150 cases (*n* = 22).
**Table S3:** Concordance of MET H‐Score ≥ 150 status between matched primary tumor and lymph node metastasis samples from the combined esophageal and gastric adenocarcinoma cohort. Cohen's κ measures agreement beyond chance (interpretation: 0.80 = almost perfect). PPA = positive percent agreement (% of primary‐positive cases also positive in lymph node metastasis); NPA = negative percent agreement (% of primary‐negative cases also negative in lymph node metastasis). Analysis uses maximum H‐Score when multiple TMA spots were available per tissue type per patient.
**Table S4:** Prognostic effect of MET 3+ status in the full esophageal adenocarcinoma cohort versus the triple negative subgroup (HER2‐negative AND Claudin 18.2‐negative AND pMMR/MSS, all evaluable, with complete covariate data). Median overall survival (Kaplan–Meier), log‐rank test, and multivariable‐adjusted hazard ratio for MET 3+ any versus negative are shown for both populations on the same complete‐case cohort. The multivariable Cox model adjusts for age (per 10 years), sex, T‐stage (T3‐4 vs. T1‐2), N‐stage (N+ vs. N0), and neoadjuvant therapy. The full cohort uses the lifelines L2‐penalized estimator (λ = 0.5; identical to manuscript Table 3); the triple‐negative subgroup model uses scikit‐survival's ridge‐penalized CoxPHSurvivalAnalysis (α = 1.0) because all 9 MET 3+ patients in this subgroup died, producing perfect separation in the censoring direction that prevented the lifelines fitter from converging. Wald CIs and *p* values for the subgroup model were computed analytically from the observed information matrix at the fitted coefficients. HR, hazard ratio; CI, confidence interval.

## Data Availability

The data supporting the findings of this study are available from the corresponding author upon reasonable request.

## References

[1] K. Crew , “Epidemiology of Upper Gastrointestinal Malignancies,” Seminars in Oncology 31, no. 4 (2004): 450–464, 10.1053/j.seminoncol.2004.04.021. 15297938

[2] C. Q. Liu , Y. L. Ma , Q. Qin , et al., “Epidemiology of Esophageal Cancer in 2020 and Projections to 2030 and 2040,” Thorac Cancer 14, no. 1 (2023): 3–11, 10.1111/1759-7714.14745. PMC9807450 36482832

[3] M. R. Sweed , D. Edmonson , and S. J. Cohen , “Tumors of the Esophagus, Gastroesophageal Junction, and Stomach,” Seminars in Oncology Nursing 25, no. 1 (2009): 61–75, 10.1016/j.soncn.2008.10.005. 19217506

[4] D. Lin , U. Khan , Goetze TO , et al., “Gastroesophageal Junction Adenocarcinoma: Is There an Optimal Management?,” American Society of Clinical Oncology Educational Book 39 (2019): e88–e95, 10.1200/EDBK_236827. 31099690

[5] S. Agarwal , M. G. Bell , L. Dhaliwal , et al., “Population Based Time Trends in the Epidemiology and Mortality of Gastroesophageal Junction and Esophageal Adenocarcinoma,” Digestive Diseases and Sciences 69, no. 1 (2024): 246–253, 10.1007/s10620-023-08126-6. PMC10926253 37914889

[6] S. E. Oh , S. Park , S. Ahn , et al., “Prognostic Significance of Esophagogastric Junction Invasion in Patients With Adenocarcinoma of the Cardia or Subcardia,” Cancers (Basel) 15, no. 6 (2023): 1656, 10.3390/cancers15061656. PMC10046536 36980541

[7] Y. J. Bang , E. Van Cutsem , A. Feyereislova , et al., “Trastuzumab in Combination With Chemotherapy Versus Chemotherapy Alone for Treatment of HER2‐Positive Advanced Gastric or Gastro‐Oesophageal Junction Cancer (ToGA): A Phase 3, Open‐Label, Randomised Controlled Trial,” Lancet 376, no. 9742 (2010): 687–697, 10.1016/S0140-6736(10)61121-X. 20728210

[8] Y. Y. Janjigian , K. Shitara , M. Moehler , et al., “First‐Line Nivolumab Plus Chemotherapy Versus Chemotherapy Alone for Advanced Gastric, Gastro‐Oesophageal Junction, and Oesophageal Adenocarcinoma (CheckMate 649): A Randomised, Open‐Label, Phase 3 Trial,” Lancet 398, no. 10294 (2021): 27–40, 10.1016/S0140-6736(21)00797-2. PMC8436782 34102137

[9] K. Shitara , F. Lordick , Y. J. Bang , et al., “Zolbetuximab Plus mFOLFOX6 in Patients With CLDN18.2‐Positive, HER2‐Negative, Untreated, Locally Advanced Unresectable or Metastatic Gastric or Gastro‐Oesophageal Junction Adenocarcinoma (SPOTLIGHT): A Multicentre, Randomised, Double‐Blind, Phase 3 Trial,” Lancet 401, no. 10389 (2023): 1655–1668, 10.1016/S0140-6736(23)00620-7. 37068504

[10] L. Trusolino and P. M. Comoglio , “Scatter‐Factor and Semaphorin Receptors: Cell Signalling for Invasive Growth,” Nature Reviews. Cancer 2, no. 4 (2002): 289–300, 10.1038/nrc779. 12001990

[11] S. L. Organ and M. S. Tsao , “An Overview of the c‐MET Signaling Pathway,” Therapeutic Advances in Medical Oncology 3, no. 1 Suppl (2011): S7–S19, 10.1177/1758834011422556. PMC3225017 22128289

[12] G. Recondo , J. Che , P. A. Jänne , and M. M. Awad , “Targeting MET Dysregulation in Cancer,” Cancer Discovery 10, no. 7 (2020): 922–934, 10.1158/2159-8290.CD-19-1446. PMC7781009 32532746

[13] E. Gherardi , W. Birchmeier , C. Birchmeier , and G. Vande Woude , “Targeting MET in Cancer: Rationale and Progress,” Nature Reviews. Cancer 12, no. 2 (2012): 89–103, 10.1038/nrc3205. 22270953

[14] R. Wang , B. Hu , Z. Pan , et al., “Antibody‐Drug Conjugates (ADCs): Current and Future Biopharmaceuticals,” Journal of Hematology & Oncology 18, no. 1 (2025): 51, 10.1186/s13045-025-01704-3. PMC12044742 40307936

[15] U.S. FDA Approves EMRELIS (Telisotuzumab Vedotin‐tllv) for Adults With Previously Treated Advanced Non‐Small Cell Lung Cancer (NSCLC) With High c‐Met Protein Overexpression. AbbVie News Center , “U.S. FDA Approves EMRELIS (Telisotuzumab Vedotin‐tllv) for Adults With Previously Treated Advanced Non‐Small Cell Lung Cancer (NSCLC) With High c‐Met Protein Overexpression. AbbVie News Center,” (2026), https://news.abbvie.com/2025‐05‐14‐U‐S‐FDA‐Approves‐EMRELIS‐TM‐telisotuzumab‐vedotin‐tllv‐for‐Adults‐With‐Previously‐Treated‐Advanced‐Non‐Small‐Cell‐Lung‐Cancer‐NSCLC‐With‐High‐c‐Met‐Protein‐Overexpression.

[16] D. R. Camidge , J. Bar , H. Horinouchi , et al., “Telisotuzumab Vedotin Monotherapy in Patients With Previously Treated c‐Met Protein‐Overexpressing Advanced Nonsquamous EGFR‐Wildtype Non‐Small Cell Lung Cancer in the Phase II LUMINOSITY Trial,” Journal of Clinical Oncology 42, no. 25 (2024): 3000–3011, 10.1200/JCO.24.00720. PMC11361350 38843488

[17] M. Sharma , J. H. Strickler , D. Sommerhalder , et al., “First‐In‐Human Study of ABBV‐400, a Novel c‐Met–Targeting Antibody‐Drug Conjugate, in Advanced Solid Tumors: Results in Colorectal Cancer,” Journal of Clinical Oncology 42, no. S16 (2024): 3515, 10.1200/JCO.2024.42.16_suppl.3515.

[18] J. H. Strickler , A. Kawazoe , J. Li , et al., “Telisotuzumab Adizutecan (ABBV‐400; Temab‐A) Monotherapy vs Trifluridine/Tipiracil Plus Bevacizumab in Patients With Refractory Metastatic Colorectal Cancer With Increased c‐Met Protein Expression: An Open‐Label, Randomized, Phase 3 Trial,” Journal of Clinical Oncology 43, no. S16 (2025): TPS3635, 10.1200/JCO.2025.43.16_suppl.TPS3635.

[19] J. H. Strickler , J. Raimbourg , F. Ghiringhelli , et al., “1439P ABBV‐400, a c‐Met Protein–Targeting Antibody‐Drug Conjugate (ADC), in Patients (Pts) With Advanced Gastric/Gastroesophageal Junction Adenocarcinoma (GEA): Results From a Phase I Study,” Annals of Oncology 35 (2024): S896, 10.1016/j.annonc.2024.08.1505.

[20] J. H. Strickler , J. Raimbourg , F. Ghiringhelli , et al., “Biomarkers of Response in Patients With Gastric/Gastroesophageal Junction Adenocarcinoma (GEA) Treated With Telisotuzumab Adizutecan,” Journal of Clinical Oncology 43, no. S4 (2025): 490, 10.1200/JCO.2025.43.4_suppl.490.

[21] K. Shitara , E. Elimova , Z. A. Wainberg , et al., “Telisotuzumab Adizutecan (ABBV‐400; Temab‐A) in Combination With Fluorouracil, Leucovorin, and Budigalimab in Locally Advanced/Metastatic Gastric, Gastroesophageal Junction, or Esophageal Adenocarcinoma (a/m GEA),” Journal of Clinical Oncology 43, no. S16 (2025): TPS4202, 10.1200/JCO.2025.43.16_suppl.TPS4202.

[22] A. H. Hölscher , P. M. Schneider , C. Gutschow , and W. Schröder , “Laparoscopic Ischemic Conditioning of the Stomach for Esophageal Replacement,” Annals of Surgery 245, no. 2 (2007): 241–246, 10.1097/01.sla.0000245847.40779.10. PMC1876980 17245177

[23] A. Quaas , A. Pamuk , S. Klein , et al., “Sex‐Specific Prognostic Effect of CD66b‐Positive Tumor‐Infiltrating Neutrophils (TANs) in Gastric and Esophageal Adenocarcinoma,” Gastric Cancer 24, no. 6 (2021): 1213–1226, 10.1007/s10120-021-01197-2. PMC8502159 34009535

[24] B. Grothey , S. I. Lyu , A. Quaas , et al., “Proteomic Characterization of MET‐Amplified Esophageal Adenocarcinomas Reveals Enrichment of Alternative Splicing‐ and Androgen Signaling‐Related Proteins,” Cellular and Molecular Life Sciences 82, no. 1 (2025): 112, 10.1007/s00018-025-05635-7. PMC11904063 40074836

[25] H. U. Schildhaus , A. M. Schultheis , J. Rüschoff , et al., “MET Amplification Status in Therapy‐naïve Adeno‐ and Squamous Cell Carcinomas of the Lung,” Clinical Cancer Research 21, no. 4 (2015): 907–915, 10.1158/1078-0432.CCR-14-0450. 25492085

[26] P. S. Plum , F. Gebauer , M. Krämer , et al., “HER2/Neu (ERBB2) Expression and Gene Amplification Correlates With Better Survival in Esophageal Adenocarcinoma,” BMC Cancer 19, no. 1 (2019): 38, 10.1186/s12885-018-5242-4. PMC6325716 30621632

[27] S. Pak , A. G. Simon , S. I. Lyu , et al., “The Expression of the Tight Junction Protein and Therapeutical Target Claudin 18.2 Is Heterogeneously Distributed Within Esophageal and Gastric Adenocarcinoma,” Scientific Reports 15 (2025): 28958, 10.1038/s41598-025-12337-4. PMC12332122 40775258

[28] A. Quaas , J. Rehkaemper , J. Rueschoff , et al., “Occurrence of High Microsatellite‐Instability/Mismatch Repair Deficiency in Nearly 2,000 Human Adenocarcinomas of the Gastrointestinal Tract, Pancreas, and Bile Ducts: A Study From a Large German Comprehensive Cancer Center,” Frontiers in Oncology 11 (2021): 569475, 10.3389/fonc.2021.569475. PMC8343401 34367937

[29] Y. Y. Janjigian , L. H. Tang , D. G. Coit , et al., “MET Expression and Amplification in Patients With Localized Gastric Cancer,” Cancer Epidemiology, Biomarkers & Prevention 20, no. 5 (2011): 1021–1027, 10.1158/1055-9965.EPI-10-1080. PMC3690490 21393565

[30] R. Erichsen , M. A. Kelsh , K. S. Oliner , et al., “Prognostic Impact of Tumor MET Expression Among Patients With Stage IV Gastric Cancer: A Danish Cohort Study,” Annals of Epidemiology 26, no. 7 (2016): 500–503, 10.1016/j.annepidem.2016.05.002. 27318530

[31] X. An , F. Wang , Q. Shao , et al., “MET Amplification Is Not Rare and Predicts Unfavorable Clinical Outcomes in Patients With Recurrent/Metastatic Gastric Cancer After Chemotherapy,” Cancer 120, no. 5 (2014): 675–682, 10.1002/cncr.28454. 24804300

[32] H. E. Lee , M. A. Kim , H. S. Lee , et al., “MET in Gastric Carcinomas: Comparison Between Protein Expression and Gene Copy Number and Impact on Clinical Outcome,” British Journal of Cancer 107, no. 2 (2012): 325–333, 10.1038/bjc.2012.237. PMC3394975 22644302

[33] S. Y. Ha , J. Lee , S. Y. Kang , et al., “MET Overexpression Assessed by New Interpretation Method Predicts Gene Amplification and Poor Survival in Advanced Gastric Carcinomas,” Modern Pathology 26, no. 12 (2013): 1632–1641, 10.1038/modpathol.2013.108. 23807774

[34] J. K. Lennerz , E. L. Kwak , A. Ackerman , et al., “MET Amplification Identifies a Small and Aggressive Subgroup of Esophagogastric Adenocarcinoma With Evidence of Responsiveness to Crizotinib,” Journal of Clinical Oncology 29, no. 36 (2011): 4803–4810, 10.1200/JCO.2011.35.4928. PMC3255989 22042947

[35] J. Zhang , L. Guo , X. Liu , W. Li , and J. Ying , “MET Overexpression, Gene Amplification and Relevant Clinicopathological Features in Gastric Adenocarcinoma,” Oncotarget 8, no. 6 (2016): 10264–10273, 10.18632/oncotarget.14382. PMC5354657 28052014

[36] Center for Drug Evaluation and Research , “FDA Grants Accelerated Approval to Telisotuzumab Vedotin‐Tllv for NSCLC With High c‐Met Protein Overexpression,” (2025), https://www.fda.gov/drugs/resources‐information‐approved‐drugs/fda‐grants‐accelerated‐approval‐telisotuzumab‐vedotin‐tllv‐nsclc‐high‐c‐met‐protein‐overexpression.

