# Supplementary material for: MET Expression in Upper Gastrointestinal Adenocarcinoma: Prevalence, Prognostic Impact, and Implications for Anti‐MET Antibody‐Drug Conjugate Therapy
Source: Int J Cancer. 2026 Jul 2;159(10):2606–18. doi: 10.1002/ijc.70634 (PMC13595460; doi:10.1002/ijc.70634)
Supplement: Supplementary file 1 [file IJC-159-2606-s001.pdf]

Supplementary Materials for:

MET Expression in Upper Gastrointestinal Adenocarcinoma: Prevalence, Prognostic Impact, and Implications for anti-MET Antibody-Drug Conjugate Therapy

Tillmann Bedau, Thomas Zander, Hans Schloesser, Hakan Alakus, Onur Mustafov, Reinhard Buettner, Christiane Bruns, Alexander Quaas

Table of Contents

Supplementary Figures ... *2*
Supplementary Figure S1 ... 2
Supplementary Figure S2 ... 3
Supplementary Figure S3 ... 4
Supplementary Figure S4 ... 5

Supplementary Tables ... *6*
Supplementary Table S1 ... 6
Supplementary Table S2 ... 7
Supplementary Table S3 ... 8
Supplementary Table S4 ... 9

Supplementary Figures

Supplementary Figure S1

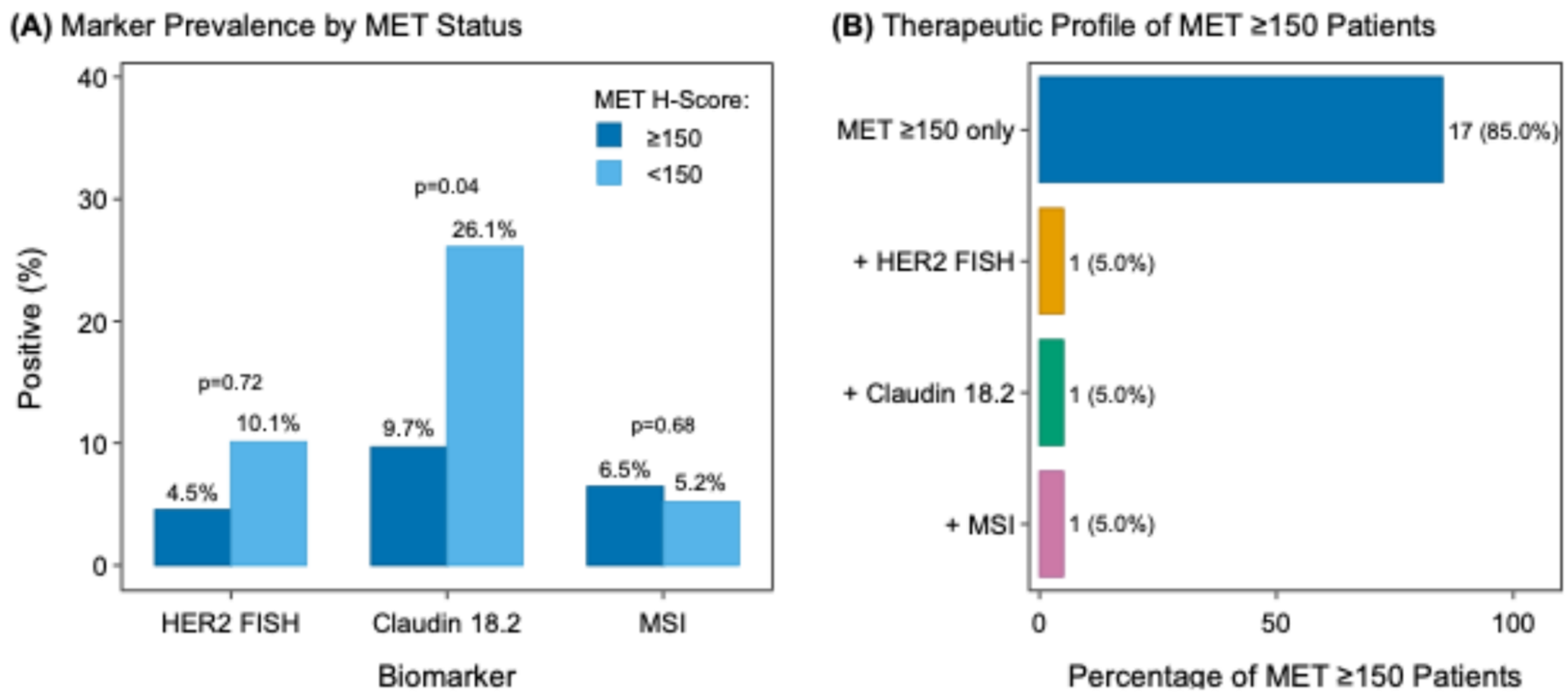

Association of MET Expression with Druggable Alterations (H-Score ≥150 Stratification). Relationship between MET protein expression and other clinically actionable biomarkers in combined esophageal and gastric adenocarcinoma using the H-Score ≥150 threshold (patient-level analysis, primary tumors only). (A) Prevalence of HER2 FISH positivity, Claudin 18.2 expression, and microsatellite instability (MSI) in patients stratified by MET H-Score (≥150 vs <150). P-values from Fisher’s exact test. (B) Therapeutic profile of MET H-Score ≥150 patients showing the proportion with no additional druggable alterations (MET ≥150 only) versus those with concurrent HER2 FISH positivity, Claudin 18.2 expression, or MSI.

Supplementary Figure S2

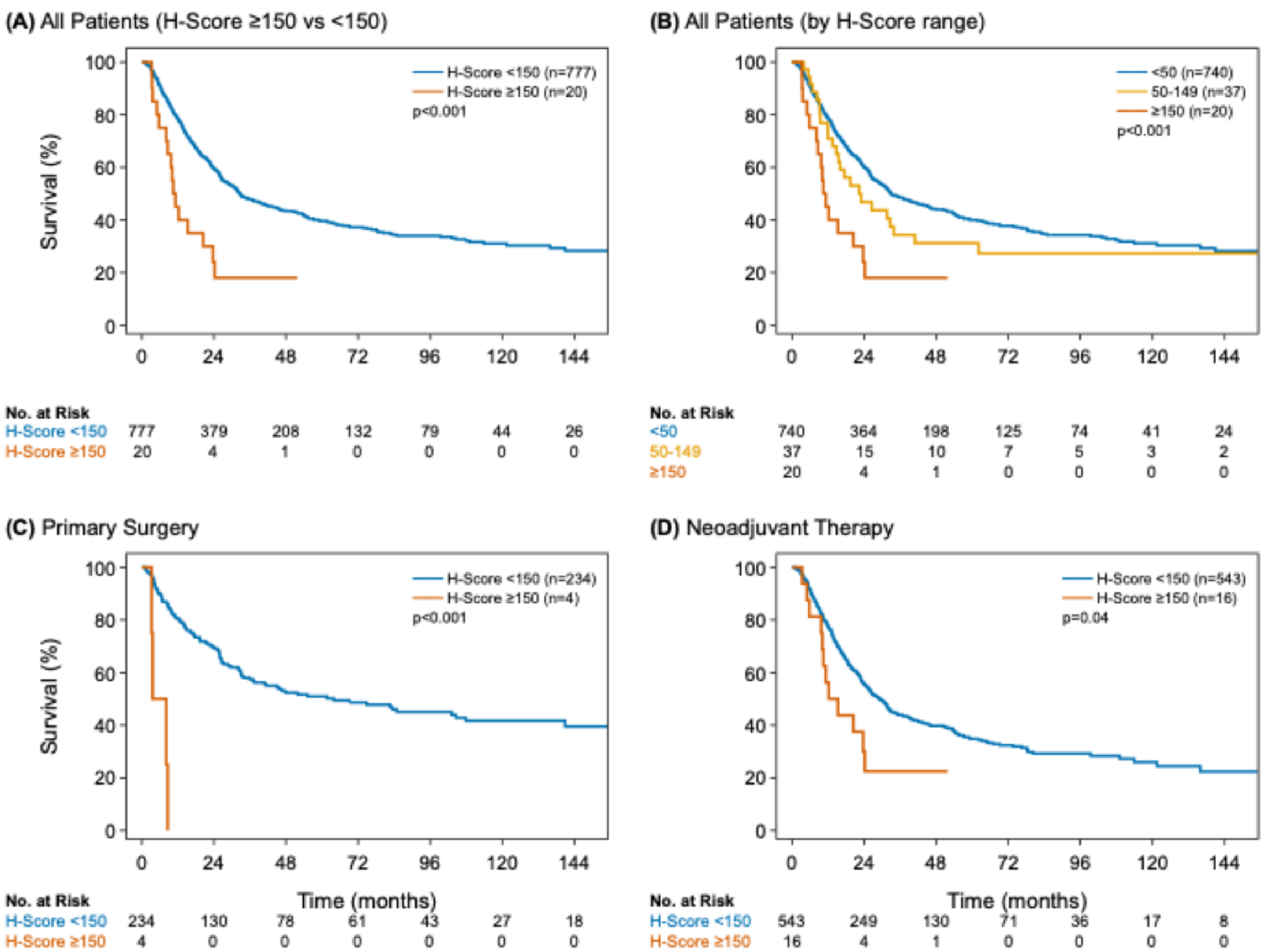

Survival Analysis by MET H-Score ≥150 Status. Kaplan-Meier curves showing overall survival in esophageal adenocarcinoma stratified by MET H-Score threshold (patient-level analysis, primary tumors only). (A) All patients comparing H-Score ≥150 versus H-Score <150. (B) All patients stratified by H-Score range (<50, 50–149, ≥150). (C) Patients treated with primary surgery without neoadjuvant therapy. (D) Patients who received neoadjuvant therapy prior to surgery. Numbers at risk are shown below each panel. Log-rank test p-values are displayed within each panel.

Supplementary Figure S3

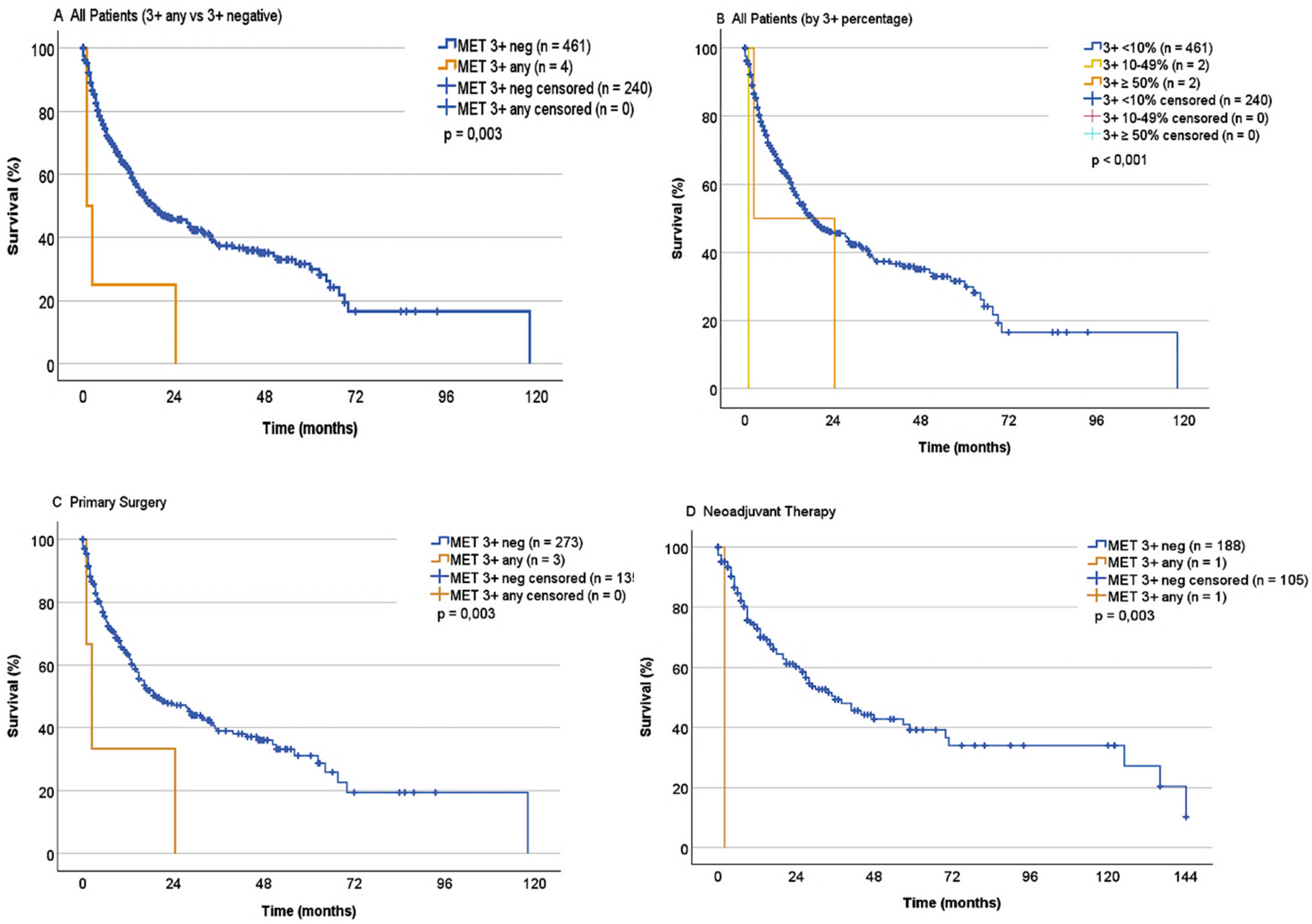

Survival Analysis by MET 3+ Status. Kaplan-Meier curves showing overall survival in gastric adenocarcinoma stratified by MET IHC 3+ expression (patient-level analysis, primary tumors only). (A) All patients comparing MET 3+ any versus MET 3+ negative. (B) All patients stratified by percentage of tumor cells with 3+ membranous staining intensity (<10%, 10–49%, ≥50%). (C) Patients treated with primary surgery without neoadjuvant therapy. (D) Patients who received neoadjuvant therapy prior to surgery. Log-rank test p-values are displayed within each panel.

Supplementary Figure S4

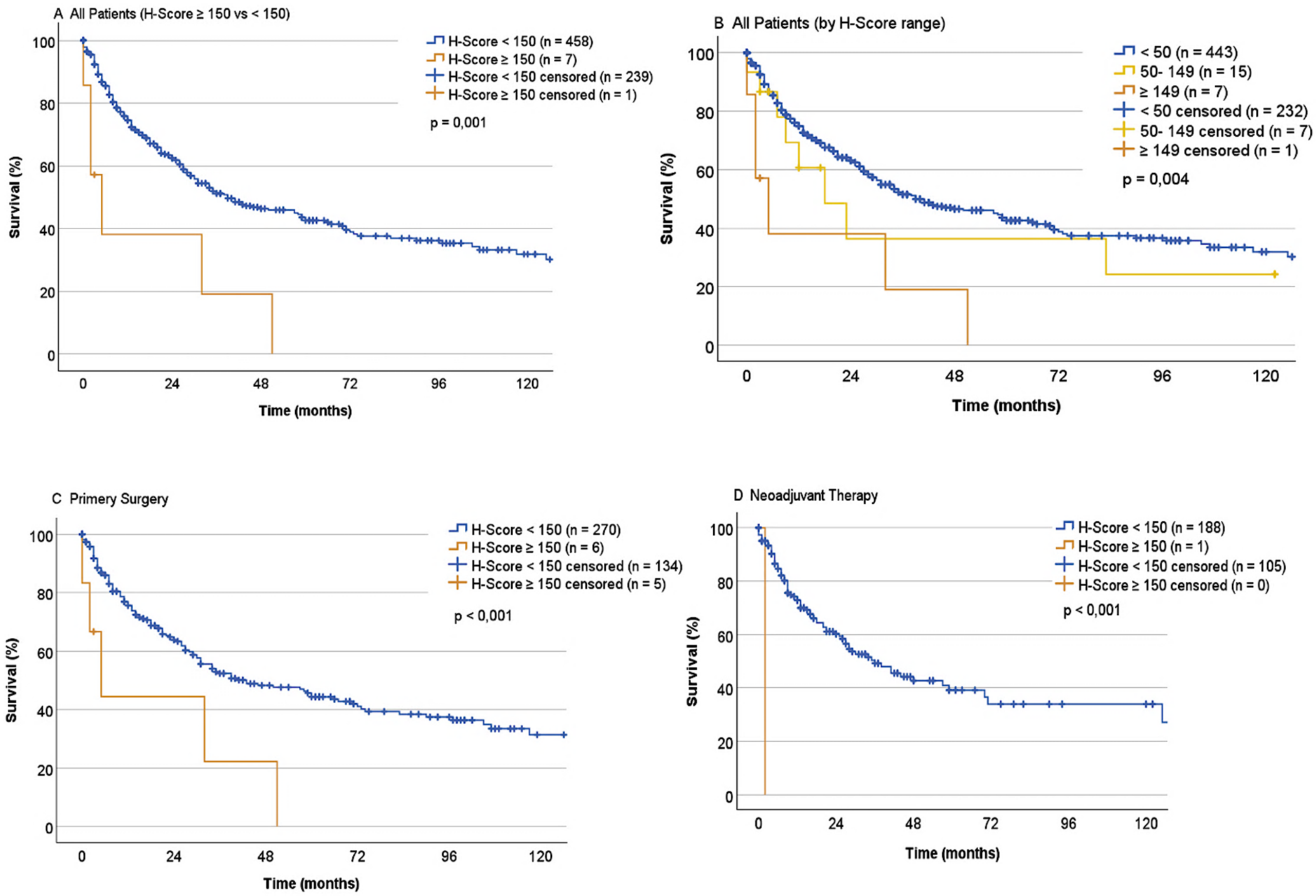

Survival Analysis by MET H-Score ≥150 Status. Kaplan-Meier curves showing overall survival in gastric adenocarcinoma stratified by MET H-Score threshold (patient-level analysis, primary tumors only). (A) All patients comparing H-Score ≥150 versus H-Score <150. (B) All patients stratified by H-Score range (<50, 50–149, ≥150). (C) Patients treated with primary surgery without neoadjuvant therapy. (D) Patients who received neoadjuvant therapy prior to surgery. Log-rank test p-values are displayed within each panel.

Supplementary Tables

Supplementary Table S1

Sensitivity analysis of H-score thresholds in the esophageal adenocarcinoma cohort. Prevalence is calculated among 984 primary-tumor patients with evaluable MET IHC (patient-level analysis, maximum H-score per patient across tissue microarray spots). MET IHC–FISH concordance is calculated among 817 patients with paired IHC and FISH data. Univariable log-rank tests were performed on the esophageal adenocarcinoma cohort with available survival data. Multivariable Cox proportional hazards regression (n=667 patients, 382 events) adjusted for age (per 10 years), sex, T-stage (T3–4 vs T1–2), N-stage (N+ vs N0), and neoadjuvant therapy, with L2 penalization (λ=0.5). The bold row indicates the primary cutoff used throughout the manuscript. The monotonic increase in odds ratios for FISH amplification and adjusted hazard ratios for overall survival across cutoffs demonstrates the robustness of the pre-specified H-score ≥150 threshold. HR, hazard ratio; CI, confidence interval.

H-score cutoff	Prevalence, n (%)	FISH amp, IHC+ vs IHC−	Odds ratio (Fisher p)	Log-rank p	Adjusted HR (95% CI; p)
**≥25**	100 (10.2%)	20/94 (21.3%) vs 13/723 (1.8%)	14.8 (p<0.001)	0.062	1.18 (0.94–1.48; p=0.16)
**≥50**	68 (6.9%)	17/64 (26.6%) vs 16/753 (2.1%)	16.7 (p<0.001)	0.011	1.28 (0.98–1.69; p=0.07)
**≥100**	33 (3.4%)	16/29 (55.2%) vs 17/788 (2.2%)	55.8 (p<0.001)	<0.001	1.69 (1.12–2.55; p=0.013)
**≥150**	**25 (2.5%)**	**14/22 (63.6%) vs 19/795 (2.4%)**	**71.5 (p<0.001)**	**<0.001**	**2.03 (1.24–3.32; p=0.005)**
**≥200**	21 (2.1%)	14/19 (73.7%) vs 19/798 (2.4%)	114.8 (p<0.001)	<0.001	2.10 (1.23–3.59; p=0.007)
**≥250**	11 (1.1%)	7/9 (77.8%) vs 26/808 (3.2%)	105.3 (p<0.001)	<0.001	2.47 (1.25–4.88; p=0.009)

Supplementary Table S2

Multivariate Cox proportional hazards regression for overall survival in esophageal adenocarcinoma using the H-Score ≥150 threshold (n=667 patients, 382 events). HR, hazard ratio; CI, confidence interval. HR >1 indicates increased risk of death. Reference categories: MET H-Score <150, female sex, T-stage T1-2, N-stage N0, no neoadjuvant therapy. Penalized maximum likelihood estimation (L2, λ=0.5) was used to ensure model convergence given the sparse MET H-Score ≥150 cases (n=22).

Variable	Category	HR	95% CI	P-value
**MET H-Score**	≥150 vs <150	2.03	1.24–3.32	0.005
**Age**	Per 10 years	1.10	1.02–1.18	0.009
**Sex**	Male vs Female	1.21	0.95–1.53	0.12
**T-stage**	T3-4 vs T1-2	1.40	1.20–1.64	<0.001
**N-stage**	N+ vs N0	1.71	0.74–3.93	0.21
**Neoadjuvant therapy**	Yes vs No	1.13	0.96–1.33	0.13

Supplementary Table S3

Concordance of MET H-Score ≥150 status between matched primary tumor and lymph node metastasis samples from the combined esophageal and gastric adenocarcinoma cohort. Cohen's κ measures agreement beyond chance (interpretation: <0 = poor, 0–0.20 = slight, 0.21–0.40 = fair, 0.41–0.60 = moderate, 0.61–0.80 = substantial, >0.80 = almost perfect). PPA = positive percent agreement (% of primary-positive cases also positive in lymph node metastasis); NPA = negative percent agreement (% of primary-negative cases also negative in lymph node metastasis). Analysis uses maximum H-Score when multiple TMA spots were available per tissue type per patient.

Statistic	H-Score ≥150
Paired cases (n)	359
Primary tumor positive	9 (2.5%)
Lymph node metastasis positive	8 (2.2%)
Both positive	3 (0.8%)
Both negative	345 (96.1%)
Primary+, LN−	6 (1.7%)
Primary−, LN+	5 (1.4%)
Cohen's κ	0.34 (fair)
PPA	33.3%
NPA	98.6%

Supplementary Table S4

Prognostic effect of MET 3+ status in the full esophageal adenocarcinoma cohort versus the triple-negative subgroup (HER2-negative AND Claudin 18.2-negative AND pMMR/MSS, all evaluable, with complete covariate data). Median overall survival (Kaplan-Meier), log-rank test, and multivariable-adjusted hazard ratio for MET 3+ any vs negative are shown for both populations on the same complete-case cohort. The multivariable Cox model adjusts for age (per 10 years), sex, T-stage (T3-4 vs T1-2), N-stage (N+ vs N0), and neoadjuvant therapy. The full cohort uses the lifelines L2-penalized estimator (λ=0.5; identical to manuscript Table 3); the triple-negative subgroup model uses scikit-survival's ridge-penalized CoxPHSurvivalAnalysis (α=1.0) because all 9 MET 3+ patients in this subgroup died, producing perfect separation in the censoring direction that prevented the lifelines fitter from converging. Wald CIs and p-values for the subgroup model were computed analytically from the observed information matrix at the fitted coefficients. HR, hazard ratio; CI, confidence interval.

Cohort	n	Events	MET 3+ (n)	Median OS, MET- vs MET+	Log-rank p	Adjusted HR (95% CI)	p
**Full esophageal cohort (Table 3)**	667	382	13	32.7 vs 9.7 mo	<0.001	2.22 (1.29–3.83)	0.004
**Triple-negative subgroup**	275	175	9	25.5 vs 9.3 mo	<0.001	4.15 (2.05–8.38)	<0.001
